# A Phylogeny-aware GWAS Framework to Correct for Heritable Pathogen Effects on Infectious Disease Traits

**DOI:** 10.1093/molbev/msac163

**Published:** 2022-08-03

**Authors:** Sarah Nadeau, Christian W Thorball, Roger Kouyos, Huldrych F Günthard, Jürg Böni, Sabine Yerly, Matthieu Perreau, Thomas Klimkait, Andri Rauch, Hans H Hirsch, Matthias Cavassini, Pietro Vernazza, Enos Bernasconi, Jacques Fellay, Venelin Mitov, Tanja Stadler, Swiss HIV Cohort Study (SHCS), I Abela, K Aebi-Popp, A Anagnostopoulos, M Battegay, E Bernasconi, DL Braun, HC Bucher, A Calmy, M Cavassini, A Ciuffi, G Dollenmaier, M Egger, L Elzi, J Fehr, J Fellay, H Furrer, CA Fux, HF Günthard, A Hachfeld, D Haerry, B Hasse, HH Hirsch, M Hoffmann, I Hösli, M Huber, CR Kahlert, L Kaiser, O Keiser, T Klimkait, RD Kouyos, H Kovari, K Kusejko, G Martinetti, de Tejada B Martinez, C Marzolini, KJ Metzner, N Müller, J Nemeth, D Nicca, P Paioni, G Pantaleo, M Perreau, A Rauch, P Schmid, R Speck, M Stöckle, P Tarr, A Trkola, G Wandeler, S Yerly

**Affiliations:** Department of Biosystems Science and Engineering, ETH Zürich, Basel, Switzerland; Swiss Institute of Bioinformatics, Lausanne, Switzerland; Precision Medicine Unit, Lausanne University Hospital and University of Lausanne, Lausanne, Switzerland; Institute of Medical Virology, University of Zurich, Zurich, Switzerland; Division of Infectious Diseases and Hospital Epidemiology, University Hospital Zurich, University of Zurich, Zurich, Switzerland; Institute of Medical Virology, University of Zurich, Zurich, Switzerland; Division of Infectious Diseases and Hospital Epidemiology, University Hospital Zurich, University of Zurich, Zurich, Switzerland; Institute of Medical Virology, University of Zurich, Zurich, Switzerland; Division of Infectious Diseases, Laboratory of Virology, Geneva University Hospital, Geneva, Switzerland; Division of Immunology and Allergy, University Hospital Lausanne, Lausanne, Switzerland; Department of Biomedicine, University of Basel, Basel, Switzerland; Department of Infectious Diseases, Bern University Hospital and University of Bern, Bern, Switzerland; Department of Biomedicine, University of Basel, Basel, Switzerland; Transplantation and Clinical Virology, Department of Biomedicine, University of Basel, Basel, Switzerland; Division of Infectious Diseases and Hospital Epidemiology, University Hospital Basel, Basel, Switzerland; Division of Infectious Diseases, University Hospital Lausanne, Lausanne, Switzerland; Division of Infectious Diseases, Cantonal Hospital St. Gallen, St. Gallen, Switzerland; Division of Infectious Diseases, Regional Hospital Lugano, Lugano, Switzerland; Swiss Institute of Bioinformatics, Lausanne, Switzerland; Precision Medicine Unit, Lausanne University Hospital and University of Lausanne, Lausanne, Switzerland; Global Health Institute, School of Life Sciences, École Polytechnique Fédérale de Lausanne, Lausanne, Switzerland; Department of Biosystems Science and Engineering, ETH Zürich, Basel, Switzerland; Swiss Institute of Bioinformatics, Lausanne, Switzerland; Department of Biosystems Science and Engineering, ETH Zürich, Basel, Switzerland; Swiss Institute of Bioinformatics, Lausanne, Switzerland

**Keywords:** genome-wide association study, infectious disease, phylogenetic mixed model, heritability

## Abstract

Infectious diseases are particularly challenging for genome-wide association studies (GWAS) because genetic effects from two organisms (pathogen and host) can influence a trait. Traditional GWAS assume individual samples are independent observations. However, pathogen effects on a trait can be heritable from donor to recipient in transmission chains. Thus, residuals in GWAS association tests for host genetic effects may not be independent due to shared pathogen ancestry. We propose a new method to estimate and remove heritable pathogen effects on a trait based on the pathogen phylogeny prior to host GWAS, thus restoring independence of samples. In simulations, we show this additional step can increase GWAS power to detect truly associated host variants when pathogen effects are highly heritable, with strong phylogenetic correlations. We applied our framework to data from two different host–pathogen systems, HIV in humans and *X. arboricola* in *A. thaliana*. In both systems, the heritability and thus phylogenetic correlations turn out to be low enough such that qualitative results of GWAS do not change when accounting for the pathogen shared ancestry through a correction step. This means that previous GWAS results applied to these two systems should not be biased due to shared pathogen ancestry. In summary, our framework provides additional information on the evolutionary dynamics of traits in pathogen populations and may improve GWAS if pathogen effects are highly phylogenetically correlated amongst individuals in a cohort.

## Introduction

A key goal of genome-wide association studies (GWAS) is to understand the genetic basis of phenotypic variation among individuals. In a typical GWAS, millions of genetic variants from across an organism’s genome are screened for statistical association with a trait of interest. Ideally, this procedure identifies variants that are located in, or are in linkage disequilibrium with, alleles that directly affect the trait. If GWAS finds a variant strongly associated with a disease trait, the gene product may be a good drug target (Okada et al. 2014). Even if no single variant has a strong association, many small associations can be aggregated into a polygenic risk score to identify susceptible individuals (Dudbridge 2013).

It is well known that GWAS can be sensitive to confounding variables. Shared ancestry among individuals, especially between close relatives, can give rise to spurious genetic correlations with a trait. Corrections for these types of population structure in human GWAS cohorts are well developed and widely accepted (Price et al. 2006; Astle and Balding 2009). More recently, analogous methods have been developed for microbial GWAS, where clonal reproduction exacerbates population structure (Power et al. 2017). Microbial GWAS-specific phylogenetic methods to account for population structure include explicitly testing for lineage-specific effects as in Earle et al. (2016) and modified association tests that account for phylogenetic relationships amongst samples as in Collins and Didelot (2018). These approaches are designed to quantify genetic effects from one organism on a trait.

In the infectious disease context, genetic effects from two organisms—the host and the pathogen—may affect an infectious disease trait. GWAS using paired host–pathogen genotype data have previously been done to elucidate the marginal and interaction effects of host and pathogen genetic variants. Methods to account for microbial population structure when testing for marginal host associations or host–pathogen interaction effects include adding the microbial kinship matrix as a random effect in a linear mixed model as in Wang et al. (2018) and using principle components derived from either this matrix or the pathogen phylogeny as covariates in a linear model as in Naret et al. (2018). These methods focus on capturing and accounting for correlations due to the pathogen phylogeny, without further investigating the nature of these correlations.

In this work, we draw from the field of phylogenetic comparative methods to propose a new two-step framework that corrects for pathogen population structure and thus satisfies the GWAS assumption of independent samples. The introduced framework relies on paired pathogen–host genotyping and is envisioned specifically for continuous-valued traits that are highly heritable from infection partner to infection partner. We hypothesized that our approach should improve GWAS power to identify host genetic variants broadly associated with disease traits.

In a first step, we fit an evolutionary model to trait data and the pathogen phylogeny. This first step provides an estimate of the correlation structure of the trait due to heritable pathogen effects. The estimate is used to remove pathogen effects on the trait. In the second step, the resulting corrected trait data are used in a GWAS with host genetic variants. The GWAS can be performed as normal under the assumption of independent samples. The main advantage of this two-step approach compared with the previously outlined methods to correct for pathogen population structure is that it generates additional information on the evolutionary dynamics of the trait in the pathogen population. The advances presented here are on the first step, whereas in the second step existing, highly optimized tools to perform GWAS association tests under a variety of models can be employed.

In the following, we describe the evolutionary model for heritable, continuous-valued infectious disease traits upon which our method is based. We derive a maximum-likelihood estimate for the pathogen part of a trait under this model. We then describe a new infectious disease GWAS framework assessing associations of the trait with host genetic variants using the maximum-likelihood estimates. In simulations, we show that this framework can improve GWAS power to detect host genetic variants that affect disease traits. Finally, we apply our framework to paired host–pathogen genotyping data from the Swiss HIV Cohort Study (SHCS) and a previously studied *Arabidosis thaliana*–*Xanthomonas arboricola* pathosystem. We show that associations with set-point viral load (spVL) and quantitative disease resistance (QDR) traits, respectively, are robust to a correction for pathogen effects.

## New Approaches

### A Statistical Model for Heritable, Continuous-valued Infectious Disease Traits

Variation in infectious disease traits like viral load or infection severity can come from several sources. These include host genetic factors, pathogen genetic factors, interaction effects between the host and the pathogen, or non-genetic factors like healthcare quality or temperature. GWAS typically stratify samples or include covariates to correct for host genetic factors or non-genetic factors that may be correlated with a trait value. This leaves pathogen genetic factors as a remaining source of correlation, since close transmission partners may be infected with very similar pathogen strains. We aim to remove this pathogen-induced correlation in the trait data prior to performing GWAS on the host genomes.

**Fig. 1.**
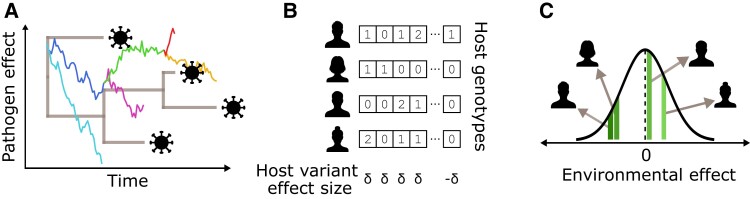
A high-level schematic of our phylogenetic Ornstein–Uhlenbeck mixed model (POUMM)-based simulation framework in the context of HIV-1 spVL. (*A*) shows how the viral effects on spVL evolve along the viral phylogeny according to an Ornstein–Uhlenbeck process. (*B*) shows how human host genetic effects are the sum of independent effects from several causal variants. Each variant can be present in 0, 1, or 2 copies. Half the variants have a positive effect of size δ and half have a negative effect of size δ. (*C*) shows how other environmental effects are independently drawn from a Gaussian distribution centered at 0. These three effects sum to the trait value for each simulated individual.

Broad-sense pathogen heritability $H^{2}$ quantifies the fraction of total variance in a trait that is “inherited” from infection partner to infection partner, that is, due to pathogen factors. To characterize $H^{2}$ and the heritable and non-heritable factors that determine infectious disease traits, we use a phylogenetic mixed model (PMM) (Housworth et al. 2004). PMMs assume continuous traits are the sum of independent heritable and non-heritable parts. In the infectious disease GWAS case, we assume the heritable part comprises pathogen genetic factors and all other factors are non-heritable. The heritable pathogen part is modeled by a random process occurring in continuous time along the branches of the pathogen phylogeny, as in figure 1*A*. The non-heritable part is modeled as Gaussian noise added to sampled individuals at the tips of the phylogeny.

PMMs have previously been applied to the study of infectious disease traits using two different types of random processes to model trait evolution. The Brownian Motion (BM) process assumes unbounded trait values, that is, the trait can attain any value. The Ornstein–Uhlenbeck (OU) process assumes trait values fluctuate around an optimal value, that is, extreme trait values are unlikely. Here, we assume the more flexible OU process as it encompasses a wider variety of evolutionary scenarios. For example, Mitov and Stadler (2018) and Bertels et al. (2018) previously showed the OU process has higher statistical support for HIV-1 spVL. This makes sense given that spVL is likely under stabilizing selection to maximize viral transmission potential (Fraser et al. 2014). The full model is called the phylogenetic Ornstein–Uhlenbeck mixed model (POUMM) and is described in detail by Mitov and Stadler (2018). Here, we review the main points relevant to our method.

Under the POUMM, the trait z is the sum of heritable genetic effects g, that is, due to the pathogen, and non-heritable “environmental” effects ϵ, that is, host genetic effects and other environmental or interaction effects:

(1) z=g+ϵ

g is a pathogen trait that evolves along the phylogeny according to an OU process. The OU process is defined by a stochastic differential equation with two terms. The first term represents a deterministic pull towards an optimal trait value and the second term represents stochastic fluctuations modeled by Brownian motion (Butler and King 2004):

(2) $$\begin{array}{rl} dg(t) & =\alpha [\theta -g(t)]\;dt+\sigma \;dW_{t}\\ g(0) & =g_{0} \end{array}$$

Here the parameter α represents selection strength towards an evolutionarily optimal value represented by parameter θ. The parameter σ measures the intensity of stochastic fluctuations in the evolutionary process. Finally, $dW_{t}$ is the Wiener process underlying Brownian motion. The OU process is a Gaussian process, meaning that g(t) is a Gaussian random variable. Assuming g(t) starts at initial value $g_{0}$ at time t=0 at the root of the phylogeny, we can write the expectation for g(t) at time t:

(3) $$E[g(t)]=g_{0}\;e^{-\alpha t}+(1-e^{-\alpha t})\theta$$

and the variance in g(t) if we were to repeat the random evolutionary process many times (Butler and King 2004):

(4) $$\mathrm{Var}[g(t)]=\frac{\sigma^{2}}{2\alpha }(1-e^{-2\alpha t})$$

g evolves independently in descendent lineages after a divergence event in the phylogeny. The covariance between g(t) in a lineage i at time $t_{i}$ and another lineage j at time $t_{j}$, $\mathrm{Cov}[g_{i}(t_{i}),g_{\;j}(t_{j})]$, increases with the amount of time between $t_{0}$ and the divergence of the two lineages, $t_{0(ij)}$, and decreases with the total amount of time the lineages evolve independently, $d_{ij}$ (Butler and King 2004):

(5) $$\mathrm{Cov}[g_{i}(t_{i}),g_{\;j}(t_{j})]=\frac{\sigma^{2}}{2\alpha }[e^{-\alpha d_{ij}}(1-e^{-2\alpha t_{0(ij)}})]$$

Next, we recall that ϵ is the non-heritable part of the trait. ϵ is modeled as a Gaussian random variable that is time- and phylogeny-independent. The expectation of ϵ is 0, meaning non-heritable effects are equally likely to raise or lower the trait from the pathogen-determined level. The parameter $\sigma_{\epsilon }^{2}$ measures the between-host variance of the non-heritable effect.

(6) $$\begin{array}{cc} & E(\epsilon )=0\\ & \mathrm{Var}(\epsilon )=\sigma_{\epsilon }^{2} \end{array}$$

Finally, broad-sense trait heritability can be calculated as the fraction of total trait variance that is heritable:

(7) $$H_{t}^{2}=\frac{\mathrm{Var}[g(t)]}{\mathrm{Var}[g(t)]+\mathrm{Var}(\epsilon )}=\frac{\left(\sigma^{2}/2\alpha )(1-e^{-2\alpha t}\right)}{(\sigma^{2}/2\alpha )(1-e^{-2\alpha t})+\sigma_{\epsilon }^{2}}$$

### Teasing Apart Pathogen and Non-pathogen Effects on a Trait

Given the assumptions of the POUMM, we can estimate a heritable pathogen effect on a trait and a corresponding non-heritable, host, and environmental effect. Here, we derive a maximum-likelihood estimate for these values for individuals in a GWAS cohort, given measured trait values and a pathogen phylogeny linking the infecting strains.

Let g(t) be a vector of g values, one for each individual in the cohort. t are the sampling times of each individual relative to the root of the phylogeny. To simplify notation, we omit the t from here on. g is a realization of a Gaussian random vector $G∼N(\mu_{\mathrm{OU}},\Sigma_{\mathrm{OU}})$. The expectation $\mu_{\mathrm{OU}}$ is defined by equation (3), the diagonal elements of the covariance matrix $\Sigma_{\mathrm{OU}}$ are defined by equation (4), and the off-diagonal elements of $\Sigma_{\mathrm{OU}}$ by equation (5). Similarly, let ϵ be a vector of the non-heritable part of the trait for each individual. ϵ is a realization of a Gaussian random vector $E∼N(0,\Sigma_{E})$, where $\Sigma_{E}$ is a diagonal matrix with diagonal elements equal to $\sigma_{\epsilon }^{2}$.

Considering that G and E are independent random vectors and that their realizations g and ϵ must sum together to equal the observed trait values z, we can write the following proportionality for the joint probability density of g and ϵ:

(8) $$f(g,\epsilon )\propto N(g;\mu_{G},\Sigma_{G})$$

where the expected value of g and the covariance matrix $\Sigma_{G}$ are defined as:

(9) $$\mathrm{Exp}(g)=\mu_{G}=\Sigma_{G}(\Sigma_{\mathrm{OU}}^{-1}\mu_{\mathrm{OU}}+\Sigma_{E}^{-1}z)$$

(10) $$\Sigma_{G}=(\Sigma_{\mathrm{OU}}^{-1}+\Sigma_{E}^{-1}{)}^{-1}$$

Proof.
(11) $$\begin{array}{rl} \;f(g,\;\epsilon ) & =f(g\;|\;\epsilon )\times f(\epsilon )\\ & =f(g)\times f(\epsilon )\\ & =N(g;\;\mu_{\mathrm{OU}},\Sigma_{\mathrm{OU}})\times N(\epsilon ;\;0,\Sigma_{E})\\ & =N(g;\;\mu_{\mathrm{OU}},\Sigma_{\mathrm{OU}})\times N(z-g;\;0,\Sigma_{E})\\ & =N(g;\;\mu_{\mathrm{OU}},\Sigma_{\mathrm{OU}})\times N(g;\;z,\Sigma_{E}) \end{array}$$

Equations (9) and (10) follow from equations 11 and 371, p. 42, section 8.1.8 “Product of Gaussian densities” in Petersen and Pedersen (2012).

Importantly, equation (9) is the maximum-likelihood estimate for g, the pathogen effect on the trait, taking into account all available information—measured trait values, the pathogen phylogeny, and inferred POUMM parameters. This estimator is an inverse-variance weighted average of measured trait (z) and information from the POUMM evolutionary model ($\mu_{\mathrm{OU}}$). In other words, g will be closer to the measured trait value if the trait is not very heritable. If the trait is highly heritable, g will be closer to the expected value under the POUMM, that is, take more information from the phylogenetic relationships between infecting strains.

Given the estimator we just derived for g, we can now estimate ϵ, the trait value without pathogen effects:

(12) $$\hat{\epsilon }=z-\mathrm{Exp}(g)$$

We will use this value to try to improve upon standard GWAS methods in infectious disease.

### A POUMM-based GWAS Framework for Infectious Disease

We propose to improve standard GWAS for infectious diseases by estimating and removing trait variability due to pathogen effects. Our new framework is as follows:

1. Sample paired host genotypes, pathogen genome sequences, and trait values from a cohort.
2. Construct a pathogen phylogeny using the pathogen genome sequences.
3. Estimate the parameters of the POUMM based on the trait values and the pathogen phylogeny. This can be done with the R package POUMM (Mitov and Stadler 2017).
4. Generate maximum-likelihood estimates for the pathogen and corresponding non-pathogen effects on the trait using equations (9) and (12).
5. Perform GWAS with only the non-pathogen effects on the trait as the response variable.

## Results

### Simulation Study

To test the theoretical best-case performance of our method, we simulated data under the POUMM and applied our framework to the simulated data. We parameterized our simulation scheme with the time-scale and other parameters of an HIV-1 outbreak in mind, with spVL as the trait of interest.

We first simulated a phylogeny of 500 tips with exponentially distributed branch lengths and mean root-to-tip time of 0.14 substitutions per site per year as in Hodcroft et al. (2014). Then, we simulated pathogen trait values g along this phylogeny using the POUMM package in R (Mitov and Stadler 2017). This part of the simulation is illustrated in figure 1*A*. For the simulation, we considered a range of pathogen heritability parameter values $H^{2}$, from 15 to 75%, and a range of selection strength parameters values α, from 0.1 to 60 time${}^{-1}$. The intensity of stochastic fluctuations parameter σ was determined based on $H^{2}$ and α [a re-arrangement of equation (4), equation given in supplementary table S1, Supplementary Material online]. As shown in supplementary figure S1, Supplementary Material online, higher α values correspond to higher σ values to maintain constant $H^{2}$ under this parameterization. For each $H^{2}$ and α value considered in the simulation, we recorded the simulated pathogen part of the trait value for each tip in the phylogeny.

We paired each tip’s simulated pathogen trait value with a simulated host trait value. Simulated hosts had 20 genome positions. We sampled alleles (0, 1, or 2) for each position from a binomial distribution with probability 0.13. Ten random positions had an effect size of 0.2 on the trait and 10 had an effect size of −0.2. This part of the simulation is illustrated in figure 1*B*. Our parameterization produced roughly normally distributed host trait values centered at 0 with variance equal to 25% of the total trait variance, which we constrained to 0.73 based on the variance in log spVL values measured by Mitov and Stadler (2018). We used 25% host heritability for spVL based on McLaren et al. (2015).

Finally, we sampled an additional random environmental effect for each tip from a normal distribution centered at 0, as illustrated in figure 1*C*. The variance of this distribution was scaled based on the pathogen heritability of the trait, from 0 (no affect) in the scenario with 75% pathogen heritability and 25% host heritability to 0.44 in the scenario with 15% pathogen heritability and 25% host heritability. Supplementary figure S2, Supplementary Material online provides a more detailed schematic of this simulation framework and supplementary table S1, Supplementary Material online gives the value or expression for each parameter.

#### Estimator Accuracy

**Fig. 2.**
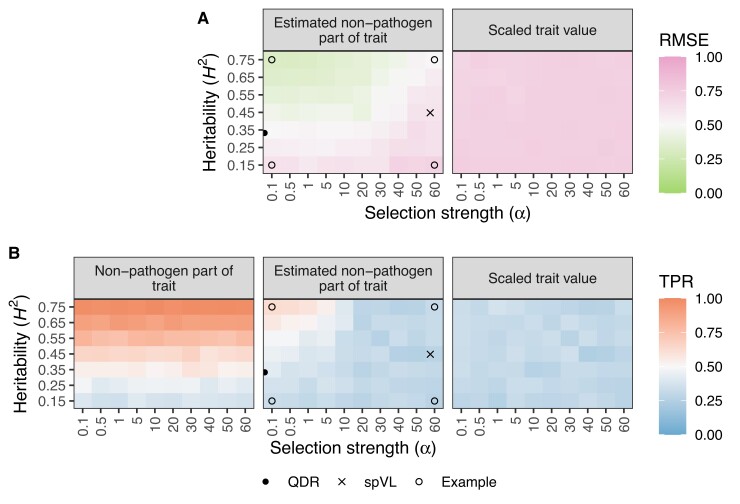
Results from the simulation study. We simulated host, pathogen, and environmental effects on a trait under the POUMM with different heritability ($H^{2}$; y-axis) and selection strength (α; x-axis) parameters. For each simulated dataset, we applied our method to estimate the non-pathogen effects and performed GWAS with these values. (*A*) shows the RMSE of our estimator (left) compared with un-corrected trait values, scaled by their mean (right) under each simulated evolutionary scenario. The RMSE is with reference to the true (simulated) host part of the trait values. Thus, more accurate estimates (lower RMSE) mean the trait value used for GWAS will be closer to the true host part of the trait value. (*B*) shows how GWAS power can improve given the true, simulated non-pathogen effect on spVL (left) and using our estimate for this value (middle) compared with using the scaled trait value (right). Each tile’s color corresponds to the average value across 20 simulated datasets of 500 samples. The points highlight specific heritability and selection strength values from the *A. thaliana*–*X. arboricola* QDR analysis, HIV-1 spVL analysis, and four simulated scenarios that are presented in more detail in figure 4.

**Fig. 3.**
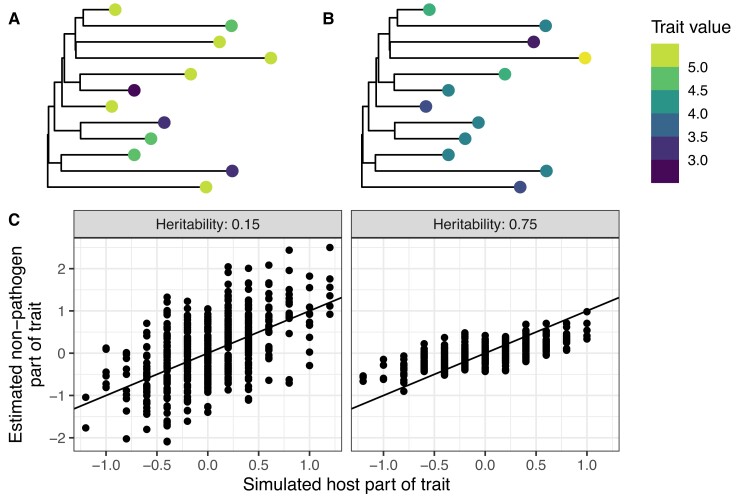
Simulated data from two evolutionary scenarios where a phylogenetic correction to trait values improve GWAS power (right side) and where it does not (left side). These examples correspond to two of the unfilled points in figure 2. (*A*,*B*) show total trait values for 12 randomly selected tips from the simulated phylogeny with pathogen heritability $H^{2}$ of 15 and 75%, respectively. Depending on the pathogen heritability, trait values are more or less correlated at clustered tips. (*C*) compares our method’s estimate for the non-pathogen part of trait values (y-axis) with true simulated host trait values (x-axis) with pathogen heritability of 15 and 75%. The solid line is the y=x line. Selection strength α was fixed to 0.1 time ${}^{-1}$ for both scenarios and all other parameters were fixed as in the full simulation study.

**Fig. 4.**
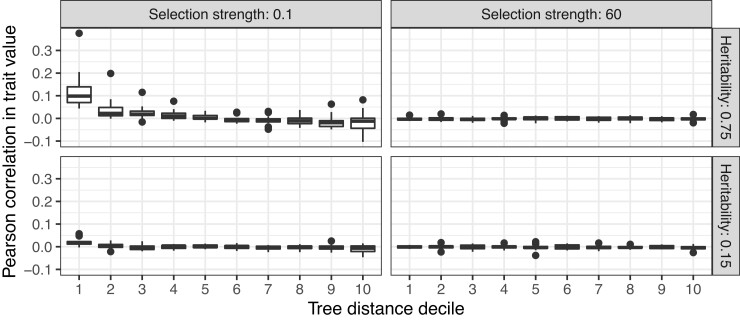
Correlations between trait values in pairs of tips in four simulated scenarios. These examples correspond to the four unfilled points in figure 2. Correlations are calculated for pairs of tips binned by phylogenetic distance (into deciles) across the 20 replicate simulations for each of the four evolutionary scenarios. Trait values are only noticeably correlated for closely clustered tips under the scenario with high pathogen heritability $H^{2}$ and low selection strength α/low stochastic fluctuations σ (upper left facet).

First, we evaluated how well our method estimated the additive host genetic effects from the simulated data. Additive host genetic effects represent an ideal (albeit unattainable) baseline for infectious disease GWAS. Figure 2*A* shows that our method incorporating phylogenetic information can more accurately estimate these value compared with the trait value. To ensure a fair comparison, we scaled trait values to have the same mean, zero, as host genetic effects so as not to bias the root mean squared error (RMSE) by a constant factor. As shown in the Supplementary material online, we can calculate the expected RMSE using the scaled trait value across scenarios in our simulation scheme because the variance in the trait due to pathogen genetic effects and environmental effects is fixed. Thus, we expect the RMSE using the scaled trait value to be 0.74 across all simulation scenarios. By incorporating phylogenetic information, we can improve upon this error in scenarios where the trait is highly heritable, under low selection pressure, and with relatively moderate stochastic fluctuations compared with outbreak duration. Figure 3 gives some intuition for how this correction works by contrasting simulated scenarios with high and low heritability and low selection strength/low stochastic fluctuations. Depending on these parameters, trait values are more or less phylogenetically correlated (see also fig. 4) and the phylogeny is more or less useful for accurately estimating the heritable pathogen and corresponding non-heritable, non-pathogen part of the trait values.

#### Theoretical GWAS Improvement

Next, we characterized the evolutionary scenarios under which our framework can actually improve GWAS power. We used the true positive rate (TPR) to evaluate the fraction of simulated causal host genetic variants we could recover as being significantly associated with the trait. We performed three different GWAS for each simulated dataset: the first represents an ideal in which we can exactly know and remove pathogen effects from trait values, the second is using our method to estimate this value and remove it, and the third represents a standard GWAS using the scaled trait value. Figure 2*B* shows that our framework can improve the TPR in simulated scenarios where selection strength <10 time${}^{-1}$ and heritability >45%. If we were able to perfectly estimate and remove pathogen effects from a trait, the TPR would increase across all values of selection strength so long as the trait is more than marginally heritable. We estimate approximately 25% to be the heritability threshold above which GWAS power is negatively impacted by pathogen effects. In summary, we show that it is theoretically possible to improve GWAS power for heritable infectious disease traits by estimating and removing pathogen effects using information from the pathogen phylogeny.

### Application to HIV-1 spVL

**Fig. 5.**
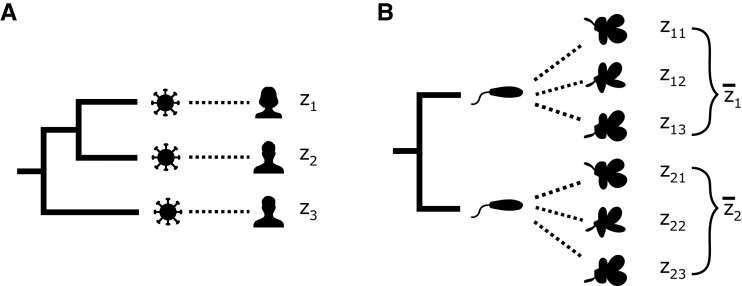
A high-level schematic of the experimental setup for the two application datasets. For (*A*) HIV-1 spVL in the Swiss HIV Cohort Study, data are paired viral and human genotypes and associated spVL measurements. We fit the POUMM to the viral phylogeny and spVL values associated with each infected individual ($z_{1},z_{2},\ldots ,z_{1493}$). For (*B*) *A. thaliana*–*X. arboricola* quantitative disease resistance (QDR) from Wang et al. (2018), data are bacterial and plant genotypes with QDR measurements for all possible combinations of pathogen and host plant strains. We fit the POUMM to the bacterial phylogeny and mean QDR calculated for each pathogen strain across all the hosts plant types (${\bar{z}}_{1},{\bar{z}}_{2},\ldots ,{\bar{z}}_{22}$).

We applied our framework to empirical data from two different host–pathogen systems with different experimental setups (fig. 5). First, we used data collected by the Swiss HIV Cohort Study (SHCS) from 1,493 individuals in Switzerland infected with HIV-1 subtype B between 1994 and 2018. The SHCS provided viral load measurements, pol gene sequences, and human genotype data for these individuals. We followed the method outlined above to estimate the pathogen and non-pathogen effects on spVL for the cohort from these data. Supplementary figure S3, Supplementary Material online shows the calculated (total) spVL values, which vary between approximately 1 and 6 log copies/mL in the cohort. We estimated spVL heritability in this cohort to be 45% (95% highest posterior density, HPD, 24–67%) and selection strength to be 58 time${}^{-1}$ (95% HPD 19–95) (supplementary fig. S4, table S2, Supplementary Material online). To put these values into the context of our simulation study, they are shown as points on figure 2. The highest expected correlation in trait values between any two tips in the HIV-1 phylogeny under the POUMM was 0.45. However, supplementary figure S5, Supplementary Material online shows that this trait is not obviously phylogenetically structured in the cohort in general, despite high heritability. Finally, supplementary figure S6, Supplementary Material online shows that the estimated non-pathogen effects on spVL correlate quite strongly with total spVL.

**Fig. 6.**
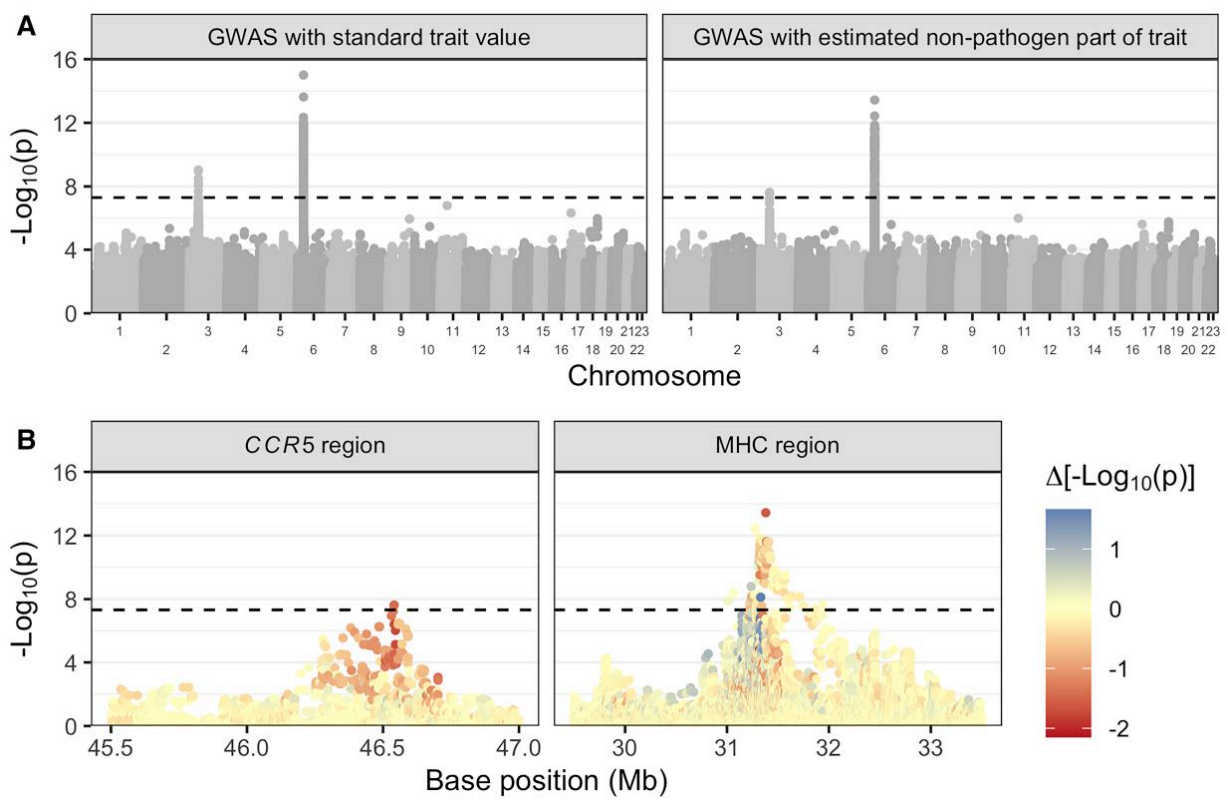
Results from comparative GWAS on HIV-1 set-point viral load (spVL) data. (*A*) shows association P values for the same host variants from the Swiss HIV cohort in GWAS with two different response variables. On the left, we used unmodified (total) spVL values. On the right, we used our estimates for the non-pathogen effects on spVL. The alternating shades correspond to different chromosomes. (*B*) compares the strength of association for variants in the *CCR5* and MHC regions between the two GWAS (positions 45.4–47 Mb on chromosome 3 and 29.5–33.5 Mb on chromosome 6 for the *CCR5* and MHC, respectively). Base positions are with reference to genome build GRCh37. The color of each point represents the difference in -log${}_{10}$P value between the two GWAS. Red means taking into account phylogenetic information decreased the strength of association and blue means it increased it. The dashed lines show genome-wide significance at $p=5\times 10^{-8}$.

We compared our proposed GWAS framework with a more standard approach by performing two different GWAS on the same SHCS human genotypes. We retained 1,392 individuals of European ancestry for the GWAS. In the (i) “GWAS with standard trait value” we used the total trait value, calculated spVL values, as the GWAS response variable. In the (ii) “GWAS with estimated non-pathogen part of trait” we used our estimates for the non-pathogen effects on spVL. Figure 6*A* shows that results are qualitatively similar between the two GWAS. Q–Q plots show the distribution of P values are very similar as well (supplementary fig. S7, Supplementary Material online). Figure 6*B* shows how the strength of association changed for some variants in the MHC and *CCR5* regions. Taking into account phylogenetic information slightly decreased association strength for most variants in the *CCR5* region. Association strength increased for some variants in the MHC, for example, SNP rs9265880 had the greatest increase in significance in the MHC region, from a P value of $3.5\times 10^{-07}$ to $7.7\times 10^{-09}$. However, the top-associated variants in the MHC and *CCR5* regions were consistent regardless of the GWAS response variable used (supplementary table S3, Supplementary Material online). Finally, table 1 shows how our GWAS results compare for the two top-associated SNPs identified by McLaren et al. (2015), who performed the largest standard GWAS for HIV spVL to date. Effect sizes are smaller with a phylogenetic correction and P values are slightly increased. We repeated the analysis using three different approximate maximum-likelihood phylogenies and these results were consistent (see Materials and Methods; supplementary table S4, Supplementary Material online). In summary, there are no clear patterns that point to new regions of association in the human genome with spVL when we take into account the pathogen phylogeny.

**Table 1.** Top Association Results from McLaren et al. (2015) Compared with Results from this Study.

Region	Variant	McLaren et al.	Standard Trait Value		Estimated Non-pathogen Part of Trait	
		P value	Effect Size	P Value	Effect Size	P Value
MHC	rs59440261	$2.0\times 10^{-83}$	− 0.4	$3.3\times 10^{-11}$	− 0.22	$2.6\times 10^{-10}$
*CCR5*	rs1015164	$1.5\times 10^{-19}$	0.15	$7.5\times 10^{-7}$	0.078	$8.5\times 10^{-6}$

Results from this study are for host variants from the SHCS in GWAS with two different response variables. “Standard trait value” means we used the unmodified (total) spVL Value and “Estimated Non-pathogen Part of Trait” Means we used our estimates for the non-pathogen effects on spVL.

### Application to the *A. thaliana*–*X. arboricola* Pathosystem

Next, we applied our method to data collected from the *A. thaliana*–*X. arboricola* pathosystem by Wang et al. (2018). Wang et al. (2018) performed a fully-crossed experiment in which they infected genetically diverse *A. thaliana* accessions with genetically diverse strains of the phytopathogenic bacteria *X. arboricola*. They scored QDR on a scale of 0 (resistant) to 4 (susceptible) for up to four infected leaves for three replicates of each *A. thaliana*–*X. arboricola* pairing. Our method requires a single-trait value per pathogen strain, so we used mean QDR calculated for each pathogen strain across all the host *A. thaliana* types (fig. 5*B*). Supplementary figure S8*A*, Supplementary Material online shows the inferred *X. arboricola* pathogen phylogeny annotated with the mean QDR trait value used for each strain. Mean QDR was generally low, varying between 0.11 for strain NL_P126 and 0.78 for strain FOR_F21. Fitting the POUMM yielded very low selection strength α and intensity of stochastic fluctuations σ parameter estimates (posterior mean 0.03 with 95% HPD 0.0–0.05 and 0.03 with 95% HPD 0.0–0.06, respectively; supplementary table S5, Supplementary Material online). These values deviated significantly from the respective priors (supplementary fig. S9, Supplementary Material online). Heritability, on the other hand, was quite uncertain (posterior mean 0.33 with 95% HPD 0.0–0.77; supplementary table S5, Supplementary Material online). The posterior mean selection strength and heritability values are also shown in the context of the simulation study as points in figure 2.

**Fig. 7.**
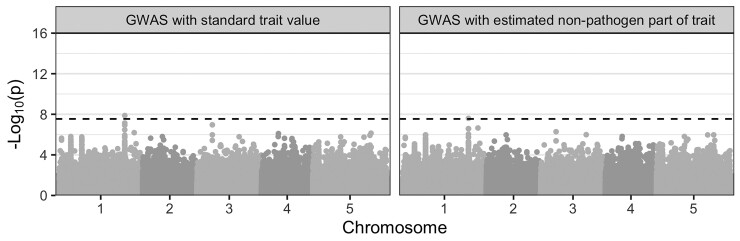
Results from comparative GWAS on *A. thaliana* QDR to *X. arboricola*. The two facets show association P values for the same host *A. thaliana* variants in GWAS with two different response variables. On the left, we used unmodified (total) QDR values for each of the 22 selected host–pathogen pairings on which these results are based. On the right, we used our estimates for the non-pathogen effects on QDR for these samples. In this case, estimated non-pathogen effects are the specific QDR for each selected host–pathogen pairing, minus mean QDR for the respective pathogen strain, calculated across all the host *A. thaliana* types. The alternating shades correspond to different chromosomes. The dashed lines show significance at significance level 0.05 with a Bonferroni correction for multiple testing.

Given the posterior mean estimates for the POUMM parameters, expected correlation in trait values between tips were very low (maximum value $3.2\times 10^{-12}$ compared with maximum value of 0.45 in the HIV-1 spVL application). Thus, the phylogeny is not very informative for a trait value correction. Indeed, the estimated pathogen part of the QDR trait calculated by our method is simply a scaling of the total QDR trait value (supplementary fig. S10, Supplementary Material online). We anyways selected 22 random host–pathogen strain pairings to perform a comparative GWAS analogous to that for HIV-1 spVL, where each host is infected with a single pathogen strain. In the first GWAS, we used the specific QDR measurement for each selected host–pathogen pairing. That is, with reference to figure 5, we selected $z_{11}$ for the first sample, $z_{23}$ for the second sample, and so on. In the second GWAS, we used our estimates for the non-pathogen effects on QDR for each pairing. Since our method did not utilize phylogenetic information in this case, the estimated non-pathogen part of the trait is simply the specific QDR for each selected host–pathogen pairing, minus mean QDR for the respective pathogen strain, calculated across all the host *A. thaliana* types. That is, with reference to figure 5, we used a scaled version of $z_{11}-{\bar{z}}_{1}$ for the first sample, $z_{23}-{\bar{z}}_{2}$ for the first sample, and so on. Figure 7 shows that results are qualitatively similar between the two GWAS, with a slight decrease in association strength for the top-associated variants. Q–Q plots show the distribution of P values are also very similar (supplementary fig. S11, Supplementary Material online). In the first, standard GWAS, one *A. thaliana* loci just exceeds the threshold for significant association after correction for multiple testing. In the second, corrected GWAS, no *A. thaliana* variants are significantly associated with QDR to *X. arboricola*.

## Discussion

In this paper, we presented a new phylogeny-aware GWAS framework to correct for heritable pathogen effects on infectious disease traits. By using information from the pathogen phylogeny, we show that it is possible to improve GWAS power to detect host genetic variants associated with a disease trait. This improved power is envisioned to contribute to a better understanding of which host factors are broadly protective against a disease versus which increase susceptibility or disease severity.

The main novelty of our approach is to estimate parameters governing the evolutionary dynamics of a trait in the pathogen population and use these estimates to correct infectious disease trait values prior to performing GWAS, thereby estimating and removing pathogen effects. In simulations, we show that when trait heritability due to shared pathogen ancestry amongst infection partners is greater than approximately 25%, GWAS power to detect host genetic variants associated with the same trait is reduced. Our method can correct for this effect in certain evolutionary scenarios by using information from the full pathogen phylogeny. Based on our simulation results, our method is anticipated to be very useful for disease traits that are highly heritable from donor to recipient and maintain a high correlation between sampled individuals. In simulations, we showed this is the case when pathogen heritability is high, selection strength is low, and trait values are not subject to strong stochastic fluctuations. In summary, cohort-level, phylogenetically structured differences in the measured trait value are necessary for our approach to outperform state-of-the-art methods.

We applied this model to two different host–pathogen systems where paired host and pathogen genetic data were generated alongside a measure of pathogen virulence. First, we fit the POUMM to spVL data from individuals living with HIV in Switzerland. We estimated HIV-1 spVL heritability to be 45% (95% HPD 24–67%) in this cohort. Compared to previous studies, this estimate is at the higher end [see Mitov and Stadler (2018) and references therein]. Also using the POUMM, Bertels et al. (2018) estimated a spVL heritability of 29% (N=2014, CI 12–46%) from the same cohort and Blanquart et al. (2017) estimated 31% (N=2,028, CI 15–43%) from a pan-European cohort. We note that our sample size (N=1,493 individuals) is smaller than in these other studies. This might be because we restricted samples based on having pol gene sequences with at least 750 non-ambiguous bases. Our aim was to reconstruct a high-quality phylogeny, since the POUMM does not account for phylogenetic uncertainty and the POUMM parameter estimates are key to our downstream trait-correction method. Although our heritability estimate is rather high, the confidence interval largely overlaps with the intervals of other studies and we note that estimating heritability per se was not our primary focus.

For comparison, we also fit the POUMM to QDR measurements from *A. thaliana* infected with the phytopathogenic bacteria *X. arboricola*. We estimated *X. arboricola* virulence heritability to be 33% (95% HPD 0–77%). Wang et al. (2018) originally estimated a QDR heritability of 44% in this dataset, falling within the wide range of our estimate. We note that Wang et al. (2018) used a linear mixed model in which the experimental unit is QDR scored on individual leaves, whereas our estimate is based on much coarser binning of QDR scores into a mean score across all leaves on all host accessions and all replicates (N=22). Furthermore, the QDR score trait values were not truly continuous (scores were measured on an integer scale from 0 to 4). Thus, these data partially violate the assumptions of the POUMM. We estimate very low selection strength for virulence in *X. arboricola*. As Wang et al. (2018) explain, *X. arboricola* strains with differing virulence can co-inhabit populations of *A. thaliana*. This might also point to low selection on *X. arboricola* virulence. Furthermore, expected correlation in virulence between related strains of *X. arboricola* was smaller than for HIV-1.

Given our estimates for trait heritability and selection strength on HIV-1 spVL and *A. thaliana* QDR to *X. arboricola*, our simulation results reveal that we cannot expect a significant improvement in GWAS power for these systems (fig. 2). Indeed, although certain pairs of samples in the HIV-1 cohort were expected to have phylogenetically correlated spVL values (maximum expected correlation between any two samples was 0.45), the overall effect on GWAS is small. For HIV-1 spVL, our phylogenetic correction slightly decreases P values for variants in CCR5 and slightly decreases some and increases other P values for variants in the MHC (fig. 6*B*). Simulations show we shouldn’t expect a net P value decrease, but our simulations represent an ideal scenario since we simulate under the POUMM. For the empirical data, un-modeled evolutionary pressures like drug treatment and host-specific HLA alleles might cause the reduced P values. However, the overall picture is consistent between the two GWAS (fig. 6*A*). For *A. thaliana* QDR to *X. arboricola*, the trait value correction does not utilize phylogenetic information because phylogenetic correlations between samples are too weak (maximum expected correlation between strains was $3.2\times 10^{-12}$). We anyways corrected QDR trait values based on average QDR for each pathogen strain across the full range of host types. Results show slight decrease in P values for the most-associated variants in this application as well, but the overall picture is consistent with previous GWAS results from Wang et al. (2018). That study found no significant *A. thaliana* variants associated with QDR using a linear mixed model jointly accounting for host genetic effects, pathogen genetic effects, and interaction effects. As with HIV-1 spVL, our results do not challenge this previous finding. Therefore, we conclude that GWAS for host determinants of HIV-1 subtype B spVL and *A. thaliana* determinants of QDR to *X. arboricola* are robust to our correction for pathogen effects.

Our method has several limitations. When POUMM parameter estimates are highly uncertain, correcting trait values based on posterior mean or maximum-likelihood parameter estimates neglects this uncertainty. Then, as in the *A. thaliana*–*X. arboricola* application, fitting the POUMM may reveal that expected phylogenetic correlations between samples are not strong enough to justify using our method to correct trait values in a GWAS. In this case, one may wish to use a linear mixed model as in Wang et al. (2018), where the pathogen effect is co-estimated as a random effect. The expected correlation structure estimated under the POUMM could be used for the covariance of the random effect, taking the phylogeny into account differently but still utilizing information from the evolutionary model. Finally, as we show here, our method is not anticipated to be useful in certain evolutionary scenarios. For instance, traits like antimicrobial resistance may be under strong selection pressure and be highly heritable. In these instances, our simulations do not point to a large improvement when adding our pre-processing step. In any case, such traits might violate the POUMM assumption that trait values vary as a random walk in continuous space if they are caused by few mutations of strong affect, meaning our approach would not apply. In this situation, one would rather account for antimicrobial resistance as a covariate in the GWAS association model.

The primary advantage of our approach is that it is complementary to previously developed methods for infectious disease GWAS. First, it provides additional information on the evolutionary dynamics of the trait in the pathogen population. Then, it is a convenient pre-processing step for GWAS because it simply produces a corrected response variable for GWAS association tests. In cases where a correction can be estimated and applied using our method, the corrected trait values are envisioned to be used in any of the previously developed GWAS models for the actual association testing (we used a linear model approach implemented in PLINK (Chang et al. 2015), though a more advanced method would be to use a linear mixed model with host ancestry as a random effect). Further, additional model complexity can be added to the GWAS association tests. For instance, our method does not account for co-infection, which might add additional variance to trait values and decrease GWAS power. In this case, one could add co-infection status as a covariate in the GWAS association test to account for this variable.

Our method relies on the freely available R package POUMM (Mitov and Stadler 2017), which scales to trees of up to 10,000 tips (Mitov and Stadler 2019). All code for the simulations and HIV spVL analysis presented in this study is available on the project GitHub at https://github.com/cevo-public/POUMM-GWAS. Future applications of our method might investigate other clinically significant disease traits and outcomes that are affected by both host and pathogen genetic factors, for instance Hepatitis B Virus-related hepatocellular carcinoma (An et al. 2018), Hepatitis C treatment success (Ansari et al. 2017), and susceptibility to or severity of certain bacterial infections, for example, Messina et al. (2016) and Donnenberg et al. (2015). Transcriptomic data have also previously been modeled as an evolving phenotype using an Ornstein–Uhlenbeck model (Rohlfs et al. 2014). Thus, one could also estimate pathogen effects on host gene expression.

In summary, we present a coherent infectious disease GWAS framework that takes the pathogen phylogeny into account when searching for host determinants of a disease trait. We further show that the pathogen phylogeny only has an impact on the GWAS outputs if heritability of the trait amongst infection partners is >25%. For the systems studied here, spVL in individuals living with HIV and QDR for *X. arboricola* infections in *A. thaliana*, the phylogenetic correction does not change GWAS results. Our findings indicate previously published GWAS results for these systems are not biased by shared evolutionary history amongst infecting pathogen strains.

## Materials and Methods

### Simulation Model

Whenever possible, we tried to parameterize our simulation model using empirical data on the spVL trait. We set the total variance in spVL to 0.73 log copies${}^{2}$ mL${}^{-2}$ based on UK cohort data (Mitov and Stadler 2018). Other studies have estimated slightly lower values though (supplementary table S6, Supplementary Material online). After allotting 25% of this variance to a host part of spVL h based on results by McLaren et al. (2015), we partitioned the remaining variance between a viral part g and an environmental part e in different ratios to assess estimator performance across a range of spVL heritabilities. h was simulated as the sum of contributions from 20 causal host genetic variants, 10 of which had an effect size of 0.2 log copies mL${}^{-1}$ and 10 of which had an effect size of −0.2 log copies mL${}^{-1}$. Host genetic variants were generated from a binomial distribution with probability p calculated such that h had the appropriate variance (see supplementary table S1, Supplementary Material online). We generated a random viral phylogeny with branch lengths on the same time scale as a previously inferred UK cohort HIV tree (Hodcroft et al. 2014) using the R package ape (Paradis and Schliep 2018). g was simulated by running an OU process along the phylogeny using the R package POUMM (Mitov and Stadler 2017) and sampling values at the tips. For the OU parameters θ and $g_{0}$, we used 4.5 log copies mL${}^{-1}$ based on previous estimates of mean spVL (supplementary table S6, Supplementary Material online). This is similar to values previously inferred for HIV (supplementary table S7, Supplementary Material online). To assess our estimator’s performance under a range of evolutionary scenarios, we co-varied the heritability $H^{2}$ and selection strength α parameters. The intensity of random fluctuations σ was determined based on these parameters (supplementary table S1 and fig. S1, Supplementary Material online). Finally, the environmental part of spVL e was generated from a normal distribution with mean 0. For a full graphical model representation of the simulation scheme, see supplementary figure S2, Supplementary Material online.

We performed GWAS on the simulated data using a linear association model as implemented in the “lm” function in R. For each simulated dataset, we performed three association tests: (i) using the true (simulated) non-pathogen part of the trait (host + environmental parts), (ii) using the estimated non-pathogen part of the trait according to the method presented in this paper, and (iii) using the total trait value, scaled by its mean. We assessed the significance of each associations at a significance level of 0.05 with a Bonferroni correction for multiple testing. For our main results (fig. 2) we simulated 20 truly associated variants, as described above. To also check the false positive rate (FPR), we re-ran the simulations with an additional 80 non-associated variants. Across all the association tests in this second simulation setup (7 $H^{2}$ levels ×10 α levels ×100 variants ×20 replicates per scenario = 140,000 association tests), FPR was 0.0005 using the true (simulated) non-pathogen part of the trait, 0.0005 using the estimated non-pathogen part of the trait, and 0.0006 using the scaled total trait value. These rates are comparable to the expected FPR of 0.0005 at significance level 0.05 corrected for 100 tests. Given the stricter correction for multiple testing in this second simulation setup, the TPR decreased significantly across all three GWAS response variables used.

### Swiss HIV-1 Data

Human genotypes, viral load measurements, and HIV-1 pol gene sequences from HIV-1 positive individuals were all collected in the context of other studies by the Swiss HIV Cohort Study (SHCS) (www.shcs.ch, Schoeni-Affolter et al. 2010; Scherrer et al. 2021). All participants were HIV-1-infected individuals 16 years or older and written informed consent was obtained from all cohort participants. The anonymized data were made available for this study after the study proposal was approved by the SHCS.

For phylogenetic inference, we retained sequences from 1,493 individuals with non-recombinant subtype B pol gene sequences of at least 750 characters and paired RNA measurements allowing for calculation of spVL, as well as five randomly chosen subtype A sequences as an outgroup. We used MUSCLE version 3.8.31 (Edgar 2004) to align the pol sequences with –maxiters 3 and otherwise default settings. We trimmed the alignment to 1505 characters to standardize sequence lengths. We used IQ-TREE version 1.6.9 (Nguyen et al. 2014) to construct an approximate maximum likelihood tree with -m GTR+F+R4 for a general time reversible substitution model with empirical base frequencies and four free substitution rate categories. Otherwise, we used the default IQ-TREE settings. After rooting the tree based on the subtype A samples, we removed the outgroup. Viral subtype was determined by the SHCS using the REGA HIV subtyping tool version 2.0 (de Oliveira et al. 2005). We calculated spVL as the arithmetic mean of viral RNA measurements made prior to the start of antiretroviral treatment. For a comparison of several different filtering methods, see supplementary figure S3, Supplementary Material online.

For GWAS, we retained data from 1,392 of the 1,493 SHCS individuals with European ancestry who were not closely related to other individuals in the cohort (supplementary table S8, Supplementary Material online). These were 227 females and 1,165 males. Ancestry was determined by plotting individuals along the three primary axes of genotypic variation from a combined dataset of SHCS samples and HapMap populations (supplementary fig. S12, Supplementary Material online). Kinship was evaluated using PLINK version 2.3 (Chang et al. 2015); we used the –king-cutoff option to exclude one from each pair of individuals with a kinship coefficient >0.09375. Initial host genotyping quality control and imputation were done as in Thorball et al. (2021). Subsequent genotyping quality control was performed using PLINK version 2.3 (Chang et al. 2015). We used the options –maf 0.01, –geno 0.01, and –hwe 0.00005 to remove variants with minor allele frequency less than 0.01, missing call rate greater than 0.05, or Hardy–Weinberg equilibrium exact test P value less than $5\times 10^{-5}$. After quality filtering, approximately 6.2 million genetic variants from the 1,392 individuals were retained for GWAS (supplementary table S9, Supplementary Material online).

### *A. thaliana*–*X. arboricola* Data

*Arabidosis thaliana* and *X. arboricola* genotyping and QDR measurements were generated by Wang et al. (2018) and are described in detail in that publication. Briefly, Wang et al. (2018) infected different *A. thaliana* host accessions with different *X. arboricola* pathogen strains in a fully-crossed experimental design. They infected up to four leaves on each of three biological replicates for each host–pathogen pairing. Then, they scored QDR for each leaf on a scale of 0 (resistant) to 4 (susceptible). We downloaded the genotype matrix with allele dosage of 33,610 SNPs for the 22 *X. arboricola* pathogen strains generated by Wang et al. (2018) from their supplementary material. We additionally downloaded a VCF file with allele dosage of 12,883,854 SNPs for the different *A. thaliana* accessions from the 1,001 Genomes project (Alonso-Blanco et al. 2016). QDR measurements were provided directly by the Wang et al. (2018) authors.

For phylogenetic inference, we used the “dist.gene” and “nj” functions from the ape package in R to construct a pairwise genetic distance matrix and then a neighbor-joining tree from the *X. arboricola* pathogen genotype matrix. The inferred tree topology (supplementary fig. S8, Supplementary Material online) closely matches the hierarchical clustering presented in (Wang et al. 2018), which was generated using the unweighted pair group method with arithmetic mean (UPGMA). Compared to UPGMA, the neighbor-joining method we used relaxes the assumptions of a strict molecular clock and sampling all at the same time-point. For the trait value to fit the POUMM, we calculated mean QDR across all leaves infected on all hosts for each *X. arboricola* strain (see fig. 5*B*) We used PLINK version 2.0 to select bi-allelic variants from the VCF file using option –max-alleles 2. We then used options –maf 0.1 and –max-maf 0.9 to remove variants with minor allele frequencies less than 0.1 as in Wang et al. (2018). After filtering, approximately 1.1 million genetic variants from *A. thaliana* were retained for GWAS (supplementary table S10, Supplementary Material online).

### POUMM Parameter Inference

We used the R package POUMM version 2.1.6 (Mitov and Stadler 2017) to infer the POUMM parameters $g_{0},\alpha ,\theta ,\sigma ,\text{and }\sigma_{e}$ from the HIV-1 and *X. arboricola* phylogenies and associated spVL and QDR trait values. The Bayesian inference method implemented in this package requires specification of a prior distribution for each parameter. For HIV-1 spVL, we used the same, broad prior distributions as in Mitov and Stadler (2018), namely: $g_{0}∼N(4.5,\;3)$, α∼Exp(0.02), θ∼N(4.5,3), $H_{\bar{t}}^{2}∼U(0,\;1)$, and $\sigma_{e}^{2}∼\mathrm{Exp}(0.02)$. For *X. arboricola* QDR, we modified the $g_{0}$ and θ priors to match the empirical mean and standard deviation of QDR trait values in the dataset: $g_{0}∼N(0.4,\;0.2)$ and θ∼N(0.4,0.2). We ran two MCMC chains for $4\times 10^{6}$ samples each with a target sample acceptance rate of 0.01 and a thinning interval of 1000 for both analyses. The first $2\times 10^{5}$ samples of each chain were used for automatic adjustment of the MCMC proposal distribution. Supplementary figures S4 and S9, Supplementary Material online show the posterior distributions for inferred parameters for HIV-1 spVL and *X. arboricola* QDR, respectively. Supplementary tables S2 and S5, Supplementary Material online give the posterior mean values used for subsequent calculations.

### Phylogenetic Trait Correction

We estimated the pathogen and non-pathogen effects on HIV-1 spVL in humans and *X. arboricola* mean QDR in *A. thaliana* using the method described in this paper. For each individual, we estimated the pathogen part of the trait value using equation (9) and the corresponding non-pathogen part using equation (12). This is implemented in the function “POUMM:::gPOUMM” in the R package POUMM. In the HIV-1 case, each sample corresponds to one HIV-1 strain with one spVL value. In the *X. arboricola* case, each sample corresponds to one *X. arboricola* strain and the mean QDR score for that strain across all host types (see fig. 5). To calculate the expected correlation in trait values between tips in the pathogen phylogeny, we used the function “covVTipsGivenTreePOUMM” in the same package. For the POUMM parameters α, σ, θ, and $\sigma_{e}$, we used the posterior mean estimates generated as described above. All the code used to implement the method is available at https://github.com/cevo-public/POUMM-GWAS.

### Association Testing

We performed two comparative GWAS for each system, using the same host genotype data across the two GWAS. For the first “GWAS with standard trait value”, we used the total (uncorrected) trait values (z) as the response variable for association testing, replicating a standard GWAS set-up. For the second “GWAS with estimated non-pathogen part of trait”, we replaced total trait values with the estimated non-pathogen component of the trait ($\hat{\epsilon }$) as the response variable. Association testing was performed using a linear association model in PLINK version 2.3 and 2.0, respectively (Chang et al. 2015) with the top 5 principle components of host genetic variation included as covariates. For the HIV-1 spVL GWAS, we additionally included sex as a covariate. The sex and principle components covariates were included to reduce residual variance and control for confounding from host population structure, respectively.

### Phylogenetic Uncertainty

Our method assumes the phylogeny accurately reflects the evolutionary relationships between pathogen strains. Previously, Hodcroft et al. (2014) observed HIV spVL heritability estimates based on pol gene sequences were robust to including or not including resistance-associated codons. Our analysis includes these codons. For the HIV application, we additionally tested the sensitivity of the inference to phylogenetic uncertainty. We inferred the phylogeny again, this time using the IQ-TREE option -wt to output all locally optimal trees. We fit the POUMM to two randomly selected trees from this set and repeated the trait correction and association testing steps using these trees and the corresponding POUMM parameter estimates.

## Supplementary Material

msac163_Supplementary_Data

## Data Availability

The simulated data underlying this article can be re-generated using the code available on the project GitHub at https://github.com/cevo-public/POUMM-GWAS. The HIV pathogen genome sequences, clinical data, and human genotypes cannot be shared publicly due to the privacy of individuals who participated in the cohort study. The data may be shared on reasonable request to the Swiss HIV Cohort Study at http://www.shcs.ch. The *X. arboricola* pathogen genotypes are available in the supplemental material of Wang et al. (2018), the *A. thaliana* host genotypes are available at https://1001genomes.org/, and the *A. thaliana*–*X. arboricola* QDR measurements are available on request to the authors of Wang et al. (2018).
