# Supplementary material for: A Phylogeny-aware GWAS Framework to Correct for Heritable Pathogen Effects on Infectious Disease Traits
Source: Mol Biol Evol. 2022 Aug 3;39(8):msac163. doi: 10.1093/molbev/msac163 (PMC9366186; doi:10.1093/molbev/msac163)
Supplement: msac163_Supplementary_Data [file msac163_supplementary_data.zip › supplement.pdf]

Supplemental Material

Expected results from simulations

Here we show the root mean square error (RMSE) of the scaled trait value for each individual, $z_i - \bar{z}$ as an estimate for the host part of the trait for each individual, h_i, should be ≈ 0.74 in our simulation scheme. First we write the expression for the RMSE:

$$RMSE = \sqrt{\frac{\sum_i^N (z_i - \bar{z} - h_i)^2}{N}} \tag{13}$$

Note that under our simulation setup, $z_i - \bar{z}$ differs from h_i due to the individual pathogen effect g_i and environmental effect e_i. So the term inside the square root equals the combined variance of these two effects:

$$RMSE = \sqrt{\sigma_g^2 + \sigma_e^2} \tag{14}$$

We can calculate the variance due to these two effects because the total variance in spVL σ_z^2, and the fraction of the total variance due to host genetic effects, σ_h^2, are fixed parameters in our simulation scheme.

$$\begin{aligned} \sigma_h^2 + \sigma_g^2 + \sigma_e^2 &= \sigma_z^2 \\ 0.25 * \sigma_z^2 + \sigma_g^2 + \sigma_e^2 &= \sigma_z^2 \\ \sigma_g^2 + \sigma_e^2 &= 0.75 * \sigma_z^2 \\ \sigma_g^2 + \sigma_e^2 &= 0.75 * 0.73 \\ \sigma_g^2 + \sigma_e^2 &= 0.55 \end{aligned} \tag{15}$$

Therefore, we can expect the RMSE for $z_i - \bar{z}$ as an estimate for h_i to be around $\sqrt{0.55} \approx 0.74$.

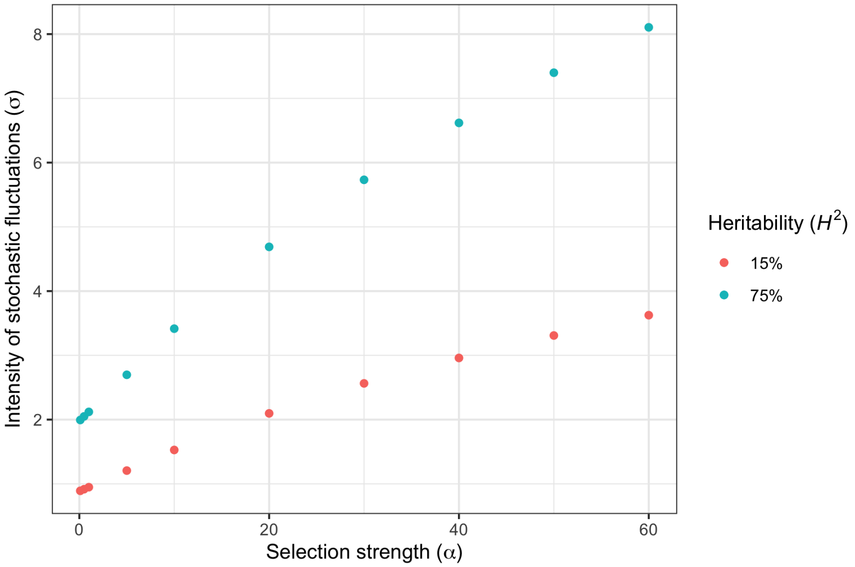

Figure S1: Relationship between the selection strength parameter α and the intensity of stochastic evolutionary fluctuations parameter σ at two different heritability (H^2) values in the simulation scheme. σ was determined as a function of α and H^2 under the POUMM (function given in Table S1).

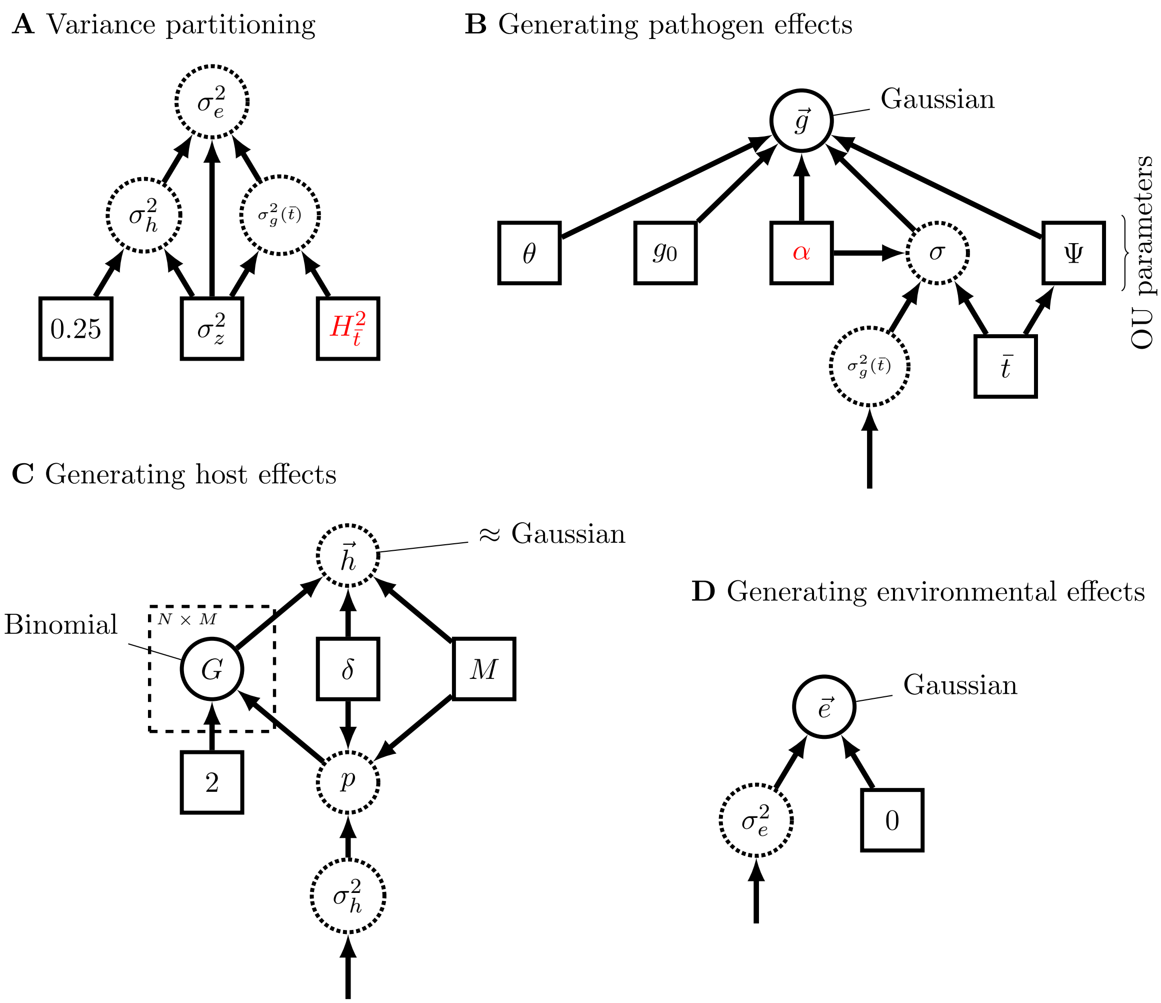

Figure S2: A graphical model representation of our simulation scheme, following the recommendations in Höhna *et al.* (2014). Variables in solid squares are constants, with the two master control variables that we vary from simulation to simulation highlighted in red. Variables in solid circles are realizations of random variables and variables in dashed circles are determined as a function of other variables. Arrows represent dependencies among variables and the dashed square represents repetition. All parameters are defined in Table S1, as well as the values or expressions used for them. (A) shows how the variance in the simulated environmental effect σ_e^2 is smaller if the master pathogen heritability value $H_{\bar{t}}^2$ is higher and vice-versa. (B) shows the OU parameters and the pathogen phylogeny, which generate the Gaussian-distributed pathogen effects. The OU parameters θ and g_0 are fixed, whereas σ is a deterministic function of the variance in the pathogen effect and the value of α. In other words, we use σ to maintain the desired pathogen heritability while varying α. (C) shows how host genotypes are drawn to generate host effects. The host genotype matrix G contains the number of copies (0, 1, or 2) for each of M causal variants with effect size δ. We assume half the variants have a positive effect and half have a negative effect. The allele frequency p for the causal variants set so that we achieve the desired variance in the host effects. (D) shows that the environmental effect is drawn from a Gaussian distribution with mean zero and variance as determined in part (A).

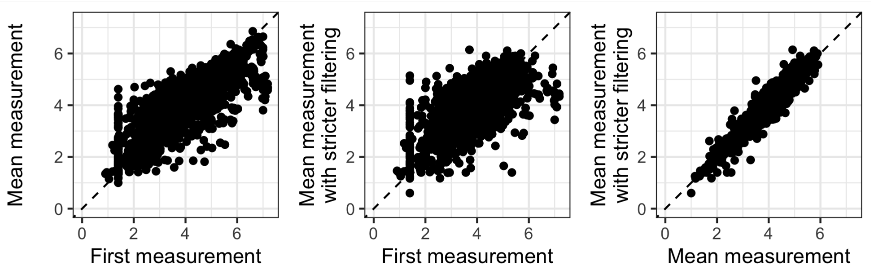

Figure S3: A comparison of different ways to calculate spVL based on viral load measurements provided by the SHCS. The stricter filtering excludes all measurements possibly < 6 months after infection and after treatment or AIDS, whereas the more lenient filtering excludes only measurements after treatment. We used the lenient filter, mean measurement values because these correlate well with the values from the stricter filter but allow us to retain many more individuals from the cohort for our study.

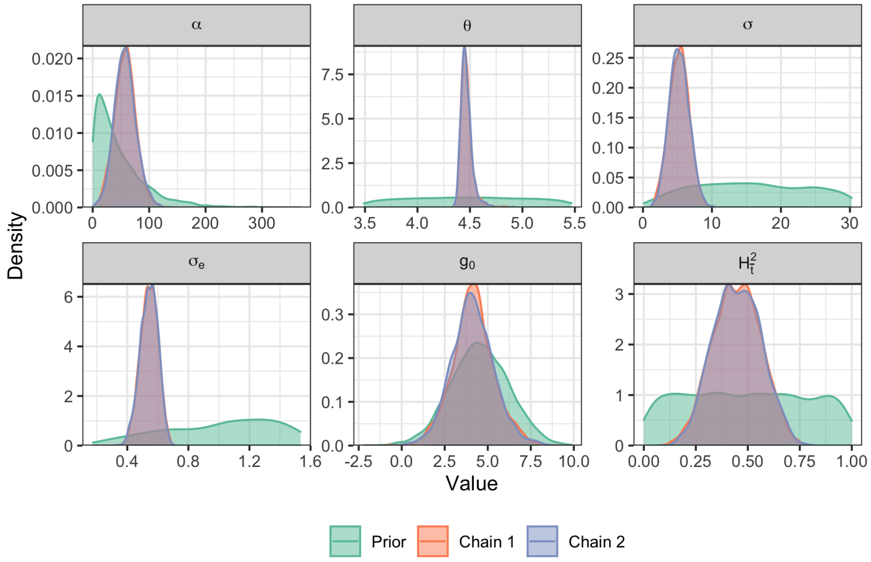

Figure S4: Posterior distributions compared to the prior for POUMM parameter estimates based on HIV-1 spVL data from the SHCS. We ran two different MCMC chains to ensure the estimates converged.

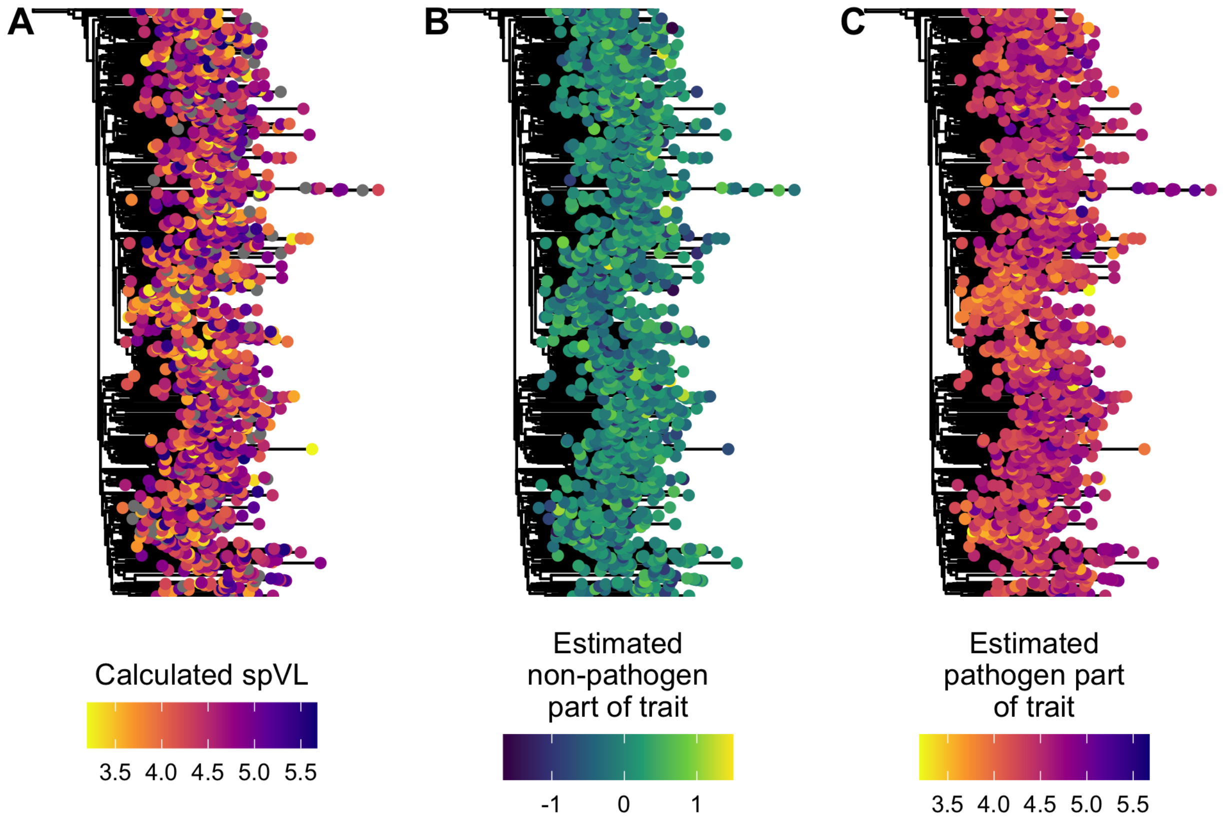

Figure S5: Inferred HIV-1 *pol* gene phylogeny with tips colored by (A) calculated spVL, (B) estimated non-pathogen effects on spVL and (C) estimated pathogen effects on spVL.

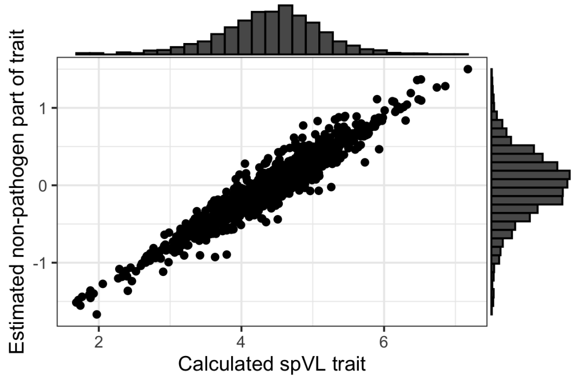

Figure S6: A comparison of measured (calculated) spVL values versus our estimated non-pathogen effect on spVL for each SHCS cohort member used in the study. The histograms show the marginal distribution of each value across the individuals.

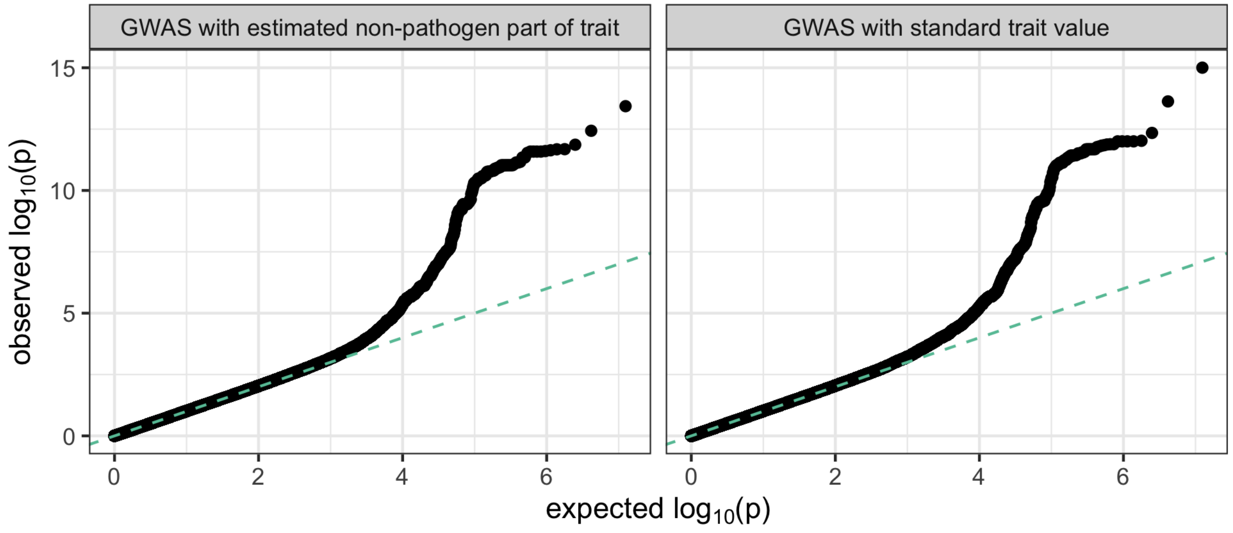

Figure S7: Quartile-quartile plots from HIV-1 spVL association tests. The dashed green line shows the y = x line.

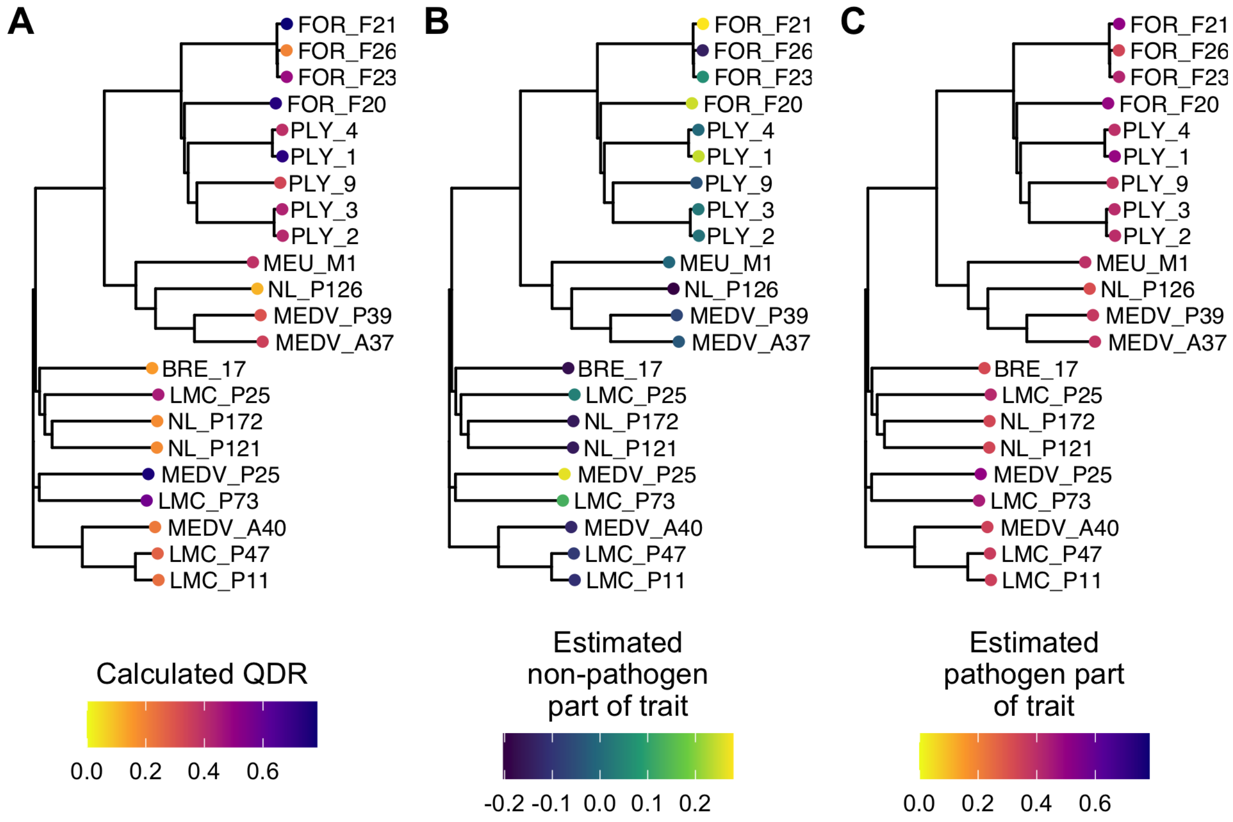

Figure S8: Inferred *X. arboricola* phylogeny with tips colored by (A) calculated QDR, (B) estimated non-pathogen effects on QDR and (C) estimated pathogen effects on QDR.

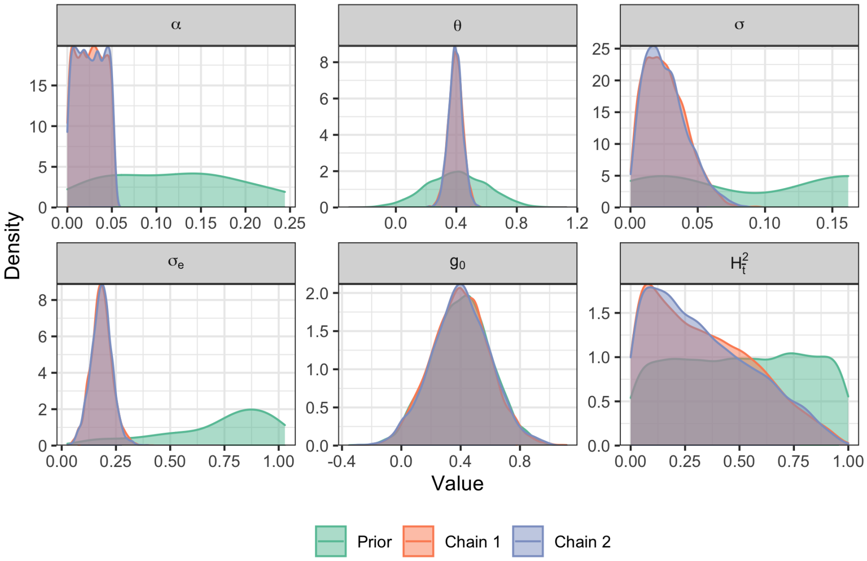

Figure S9: Posterior distributions compared to the prior for POUMM parameter estimates based on *A. thaliana*-*X. arboricola* data. We ran two different MCMC chains to ensure the estimates converged.

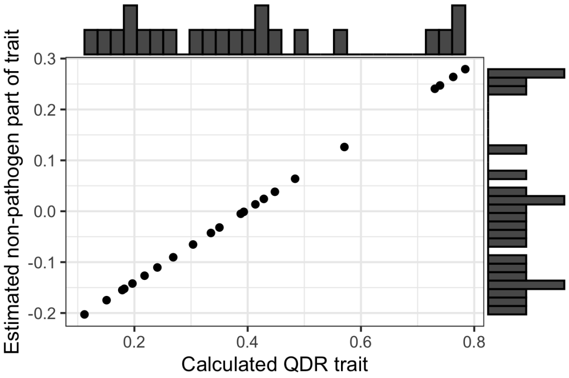

Figure S10: A comparison of measured (calculated) mean QDR values across all *A. thaliana* accession pairings and replicates versus our estimated non-pathogen effect on mean QDR for each *X. arboricola* pathogen strain. The histograms show the marginal distribution of each value across the strains. The Pearson correlation coefficient is 1.

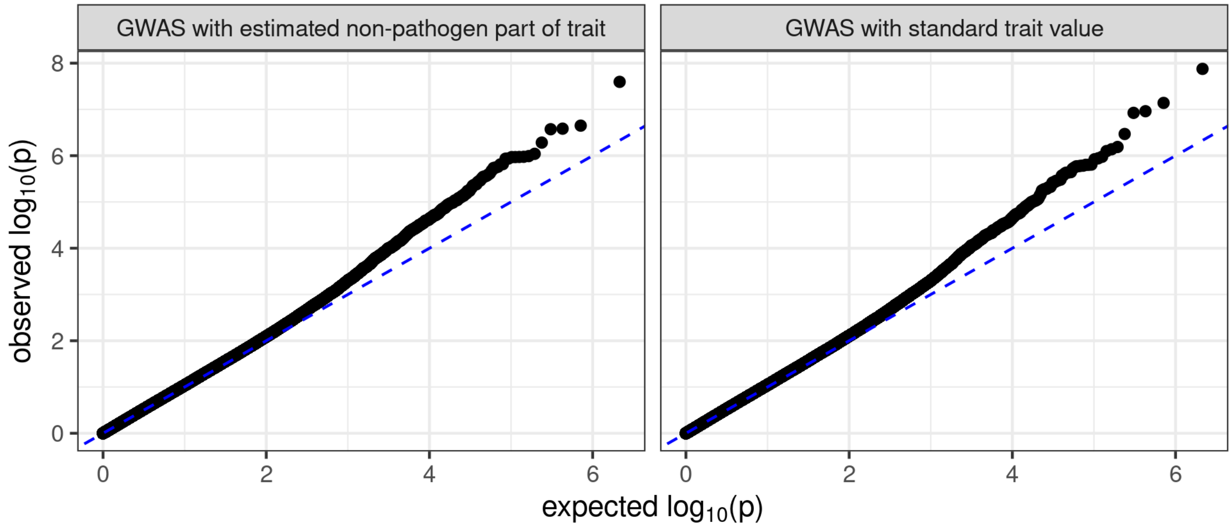

Figure S11: Quartile-quartile plots from *A. thaliana*-*X. arboricola* QDR association tests. The dashed blue line shows the y = x line.

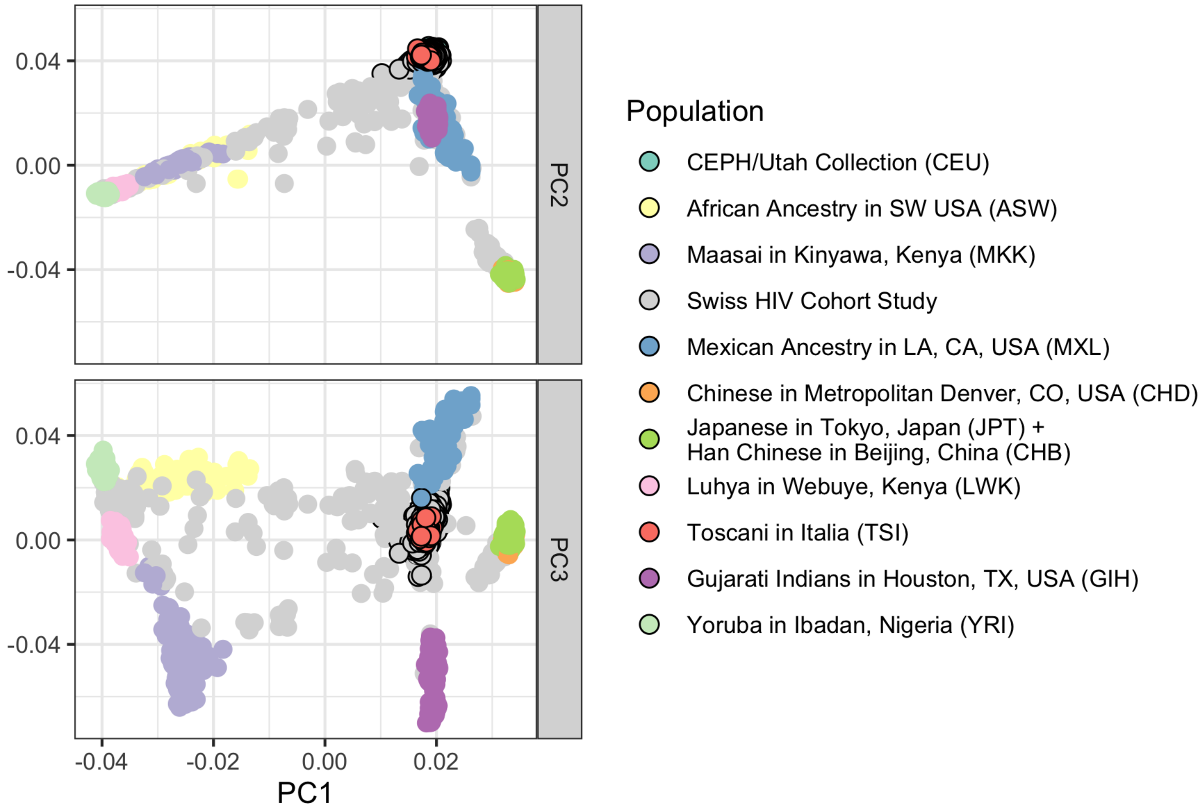

Figure S12: SHCS individuals and HapMap3 individuals plotted along the top three principle components of genetic variation. Points with black borders are within the thresholds used to select individuals of likely European ancestry.

Table S1: Simulation model parameters. For a full graphical model representation of the simulation scheme, including how these parameters are related, see Figure S2.

Variable	Expression	Definition
σ_z^2	0.73 log copies^2/mL^2	Total spVL variance
H_h^2	0.25	Host heritability of spVL
$H_{\bar{t}}^2$	varied	Pathogen heritability of spVL at $\bar{t}$
σ_h^2	$\sigma_h^2 = 0.25 * \sigma_z^2$	Variance in host part of spVL
$\sigma_g^2(\bar{t})$	$\sigma_g^2(\bar{t}) = H_{\bar{t}}^2 * \sigma_z^2$	Variance in pathogen part of spVL at $\bar{t}$
σ_e^2	$\sigma_e^2 = \sigma_z^2 - \sigma_g^2 - \sigma_h^2$	Variance in environmental part of spVL
$\bar{t}$	0.14 substitutions site^{-1} yr^{-1}	Mean root-tip time in pathogen phylogeny
$\boldsymbol{g}$	$\boldsymbol{g} \sim Norm(\boldsymbol{\mu}_{OU}, \boldsymbol{\Sigma}_{OU})$	Pathogen part of spVL for all individuals
θ	4.5 log copies/mL	Optimal spVL value
g_0	4.5 log copies/mL	g at the root of the phylogeny
α	varied	Selection strength of OU process
σ	$\sigma = \sqrt{\frac{2\alpha\sigma_g^2(\bar{t})}{1-exp(-2\alpha\bar{t})}}$	Time-unit standard deviation of OU process
Ψ	branch lengths $\sim Exp(\bar{t})$	Pathogen phylogeny
h_i	$h_i = \delta\sum_{j=1}^{j=M/2} G_{ij} - \delta\sum_{j=M/2}^{j=M} G_{ij}$	Host part of spVL for individual i
$G_{N\times M}$	$G_{ij} \sim Binom(2,p)$ $\forall i \in 1...N, \forall j \in 1...M$	Host genotype matrix
p	$p = \frac{1}{2} - \sqrt{\frac{1}{4} - \frac{H_h^2\sigma_z^2}{2\delta^2 M}}$	Host variant allele frequency
δ	0.2	Host variant effect size
M	20	Number of causal host variants
e_i	$e_i \sim Norm(0, \sigma_e^2)$	Environmental part of spVL for individual i
N	500	Number of simulated samples

Table S2: POUMM parameter estimates for spVL based on SHCS data. HPD = Highest posterior density.

Parameter	Posterior mean	95% HPD
g_0	4.23	(1.72, 6.71)
θ	4.47	(4.37, 4.58)
σ	5.25	(2.37, 7.9)
α	57.65	(19.49, 95.2)
σ_ϵ	0.54	(0.43, 0.65)
$H_{\bar{t}}^2$	0.45	(0.24, 0.67)

Table S3: Effect size and p-values from the top most strongly associated variants in the *CCR5* and MCR regions from each of the two GWAS performed in our study. "Standard" means the GWAS with standard spVL trait values and "Corrected" means the GWAS with the estimated non-pathogen part of the trait. Entries above the dividing line are the top-associated variants from the "Standard" GWAS and entries below the dividing line are the top-associated variants from the "Corrected" GWAS. Many entries overlap between the two.

Region	Position	Variant	Standard effect size	Standard p-value	Corrected effect size	Corrected p-value
CCR5	46531144	rs9845968	-0.16	5.6×10^{-9}	-0.083	1.2×10^{-7}
CCR5	46537849	rs867620	-0.16	3.2×10^{-9}	-0.085	6×10^{-8}
CCR5	46539864	rs11130092	-0.16	1.1×10^{-9}	-0.087	2.6×10^{-8}
CCR5	46540932	rs10865942	-0.16	8.4×10^{-9}	-0.081	4×10^{-7}
CCR5	46541147	rs7430431	-0.17	9.2×10^{-10}	-0.088	2.3×10^{-8}
MHC	31274380	rs9264942	-0.21	4.5×10^{-13}	-0.12	3.7×10^{-13}
MHC	31321919	rs1055821	-0.33	9.4×10^{-13}	-0.19	1.4×10^{-12}
MHC	31380034	rs112243036	-0.32	9.9×10^{-16}	-0.17	3.7×10^{-14}
MHC	31391401	rs4418214	-0.34	2.4×10^{-14}	-0.18	2.5×10^{-12}
MHC	31400137	rs138130755	-0.46	1×10^{-12}	-0.26	2.6×10^{-12}
MHC	31400705	rs138117378	-0.46	1×10^{-12}	-0.26	2.6×10^{-12}
MHC	31402358	rs148792134	-0.46	1×10^{-12}	-0.26	2.6×10^{-12}
MHC	31409677	rs140991764	-0.46	1×10^{-12}	-0.26	2.6×10^{-12}
CCR5	46531144	rs9845968	-0.16	5.6×10^{-9}	-0.083	1.2×10^{-7}
CCR5	46537849	rs867620	-0.16	3.2×10^{-9}	-0.085	6×10^{-8}
CCR5	46539864	rs11130092	-0.16	1.1×10^{-9}	-0.087	2.6×10^{-8}
CCR5	46541147	rs7430431	-0.17	9.2×10^{-10}	-0.088	2.3×10^{-8}
CCR5	46556835	rs6808142	0.15	8.3×10^{-8}	0.082	3.3×10^{-7}
MHC	31274380	rs9264942	-0.21	4.5×10^{-13}	-0.12	3.7×10^{-13}
MHC	31321919	rs1055821	-0.33	9.4×10^{-13}	-0.19	1.4×10^{-12}
MHC	31367874	rs111281598	-0.37	1.5×10^{-12}	-0.22	2.1×10^{-12}
MHC	31376266	rs73400361	-0.37	1.4×10^{-12}	-0.22	2.1×10^{-12}
MHC	31380034	rs112243036	-0.32	9.9×10^{-16}	-0.17	3.7×10^{-14}

Table S4: Results comparison using three different approximate maximum likelihood HIV-1 phylogenies (see Materials and Methods).

	Estimated non-pathogen part of trait main tree		Estimated non-pathogen part of trait tree 2		Estimated non-pathogen part of trait tree 3	
Variant	Effect size	p-value	Effect size	p-value	Effect size	p-value
rs59440261	-0.22	2.6×10^{-10}	-0.22	1.5×10^{-10}	-0.24	1.4×10^{-10}
rs1015164	0.078	8.5×10^{-6}	0.076	9.5×10^{-6}	0.083	9×10^{-6}

Table S5: POUMM parameter estimates for QDR based on *A. thaliana*-*X. arboricola* data. HPD = Highest posterior density.

Parameter	Posterior mean	95% HPD
g_0	0.40	(0.01, 0.78)
θ	0.39	(0.30, 0.49)
σ	0.03	(0.0, 0.06)
α	0.03	(0.0, 0.05)
σ_ϵ	0.18	(0.08, 0.27)
H_t^2	0.33	(0.0, 0.77)

Table S6: Summary statistics for log spVL in previously sampled populations. $\bar{z}$ is average spVL (log copies/mL) and σ_z^2 is variance in measured spVL (log copies^2/mL^2). Values from (Blanquart *et al.*, 2017; Mitov and Stadler, 2018) are empirical; values from (Bonhoeffer *et al.*, 2015) were estimated by fitting a normal distribution to the data.

Measurement	Value	Reference
$\bar{z}$	≈ 4.5	Mitov and Stadler (2018)
$\bar{z}$	4.4	Blanquart *et al.* (2017)
$\bar{z}$	≈ 4.5	Bonhoeffer *et al.* (2015)
σ_z^2	0.73	Mitov and Stadler (2018)
σ_z^2	0.50	Blanquart *et al.* (2017)
σ_z^2	≈ 0.5	Bonhoeffer *et al.* (2015)

Table S7: POUMM parameter estimates for spVL from previous studies.

Parameter	Value (Uncertainty)	Reference	Notes
g_0	5.54 (4.04 - 7.25)	Mitov and Stadler (2018)	8,483 UK HIV cohort individuals, *pol* tree
θ	4.45 (4.41 - 4.49)	Mitov and Stadler (2018)	
θ	4.0 (1.6 - 4.))	Bertels *et al.* (2018)	3,036 SHCS individuals, *pol* tree
θ	4.1 (3.5 - 4.9)	Blanquart *et al.* (2017)	1,581 subtype B individuals from Europe, whole genome tree
α	28.78 (16.64 - 46.93)	Mitov and Stadler (2018)	
α	32.7 (0.03 - 57.6)	Bertels *et al.* (2018)	
α	7.6 (1.2 - 10)	Blanquart *et al.* (2017)	**limited α to ≤ 10
σ	2.97 (1.95 - 4.37)	Mitov and Stadler (2018)	
σ	1.3 (0.66 -1.87)	Blanquart *et al.* (2017)	
σ_e	0.77 (0.73, 0.8)	Mitov and Stadler (2018)	
σ_e	0.61 (0.54, 0.65)	Blanquart *et al.* (2017)	

Table S8: Number of samples for HIV-1 spVL GWAS after sequential filtering steps.

Sample filter	Number of samples remaining
Subtype B pol sequences	1516
With paired spVL measurement	1516
$>$ 750 characters in sequence	1493
Individual is of European ancestry	1396
Kinship coefficient $>$ 0.09375	1392

Table S9: Number of variants for HIV-1 spVL GWAS after sequential filtering steps.

Variant filter	Number of variants remaining
Raw data	76979521
Missing genotype rate $>$ 0.05	11590002
Hardy-Weinburg exact test p-value $< 5 \times 10^{-5}$	11589246
Minor allele frequency $<$ 0.01	6228626

Table S10: Number of variants for *A. thaliana* QDR GWAS after sequential filtering steps. The last entry lists variants without GWAS p-values because PLINK assessed the correlation between predictor variables (the variant and the top 5 principle components of host genetic variation are predictors) to be too strong. This did not occur in the HIV-1 spVL GWAS.

Variant filter	Number of variants remaining
Raw data	12883854
Bi-allelic variants	11769920
Minor allele frequency < 0.1	1743952
NA p-value (too-high covariate correlation)	1070541
